# Assessing Agricultural Livelihood Vulnerability to Climate Change in Coastal Bangladesh

**DOI:** 10.3390/ijerph16224552

**Published:** 2019-11-18

**Authors:** Muhammad Ziaul Hoque, Shenghui Cui, Lilai Xu, Imranul Islam, Jianxiong Tang, Shengping Ding

**Affiliations:** 1 Key Laboratory of Urban Environment and Health, Institute of Urban Environment, Chinese Academy of Sciences, Xiamen 361021, China; mziahoque.aer@bsmrau.edu.bd (M.Z.H.); islam@iue.ac.cn (I.I.); jxtang@iue.ac.cn (J.T.); spding@iue.ac.cn (S.D.); 2 International School, University of Chinese Academy of Sciences, Beijing 100049, China; 3 Xiamen Key Lab of Urban Metabolism, Institute of Urban Environment, Chinese Academy of Sciences, Xiamen 361021, China; 4 Department of Agricultural Extension and Rural Development, Bangabandhu Sheikh Mujibur Rahman Agricultural University, Gazipur 1706, Bangladesh

**Keywords:** climate change, agriculture vulnerability, spatially heterogeneous, adaptation decision, coastal Bangladesh

## Abstract

The adverse impacts of climate change exert mounting pressure on agriculture-dependent livelihoods of many developing and developed nations. However, integrated and spatially specific vulnerability assessments in less-developed countries like Bangladesh are rare, and insufficient to support the decision-making needed for climate-change resilience. Here, we develop an agricultural livelihood vulnerability index (ALVI) and an integrated approach, allowing for (i) mapping out the hot spots of vulnerability distribution; (ii) identifying key factors of spatially heterogeneous vulnerability; and (iii) supporting intervention planning for adaptation. This study conceptualized vulnerability as a function of exposure, sensitivity, and adaptive capacity by developing a composite index from a reliable dataset of 64 indicators comprising biophysical, agro-ecological, and socioeconomic variables. The empirical studies of coastal Bangladesh revealed that Bhola, Patuakhali, and Lakshmipur districts, around the mouth of the deltaic Meghna estuaries, are the hot spot of vulnerability distribution. Furthermore, the spatially heterogeneous vulnerability was triggered by spatial variation of erosion, cyclones, drought, rain-fed agriculture, land degradation, soil phosphorus, crop productivity, sanitation and housing condition, infant mortality, emergency shelters, adoption of agro-technology. The integrated approach could be useful for monitoring and evaluating the effectiveness of adaptation intervention by substituting various hypothetical scenarios into the ALVI framework for baseline comparison.

## 1. Introduction 

Agriculture is the primary means of livelihood for 2.5 billion people in the world [1] and contributes 26% of GDP (gross domestic production) in the economy of many low-income developing countries [2]. However, climate change is a global driver adversely affecting the sustainability of the agricultural production system through increased variability in temperature and rainfall, and the frequency and intensity of extreme weather events. The effects are expected to be many-fold: such as altering crop pest infestation and disease outbreaks; crop failure; yield reduction; loss of fish biodiversity; and higher livestock mortality [3,4].

The coastal zones encompass only 2% of the earth’s surface but accommodate over 10% of the global population [5]. The Intergovernmental Panel on Climate Change (IPCC) has projected that sea-level rise will continue throughout the 21st century and beyond that [6], which in turn will adversely affect the coastal region of the tropical and sub-tropical developing countries because of their over-dependence on natural resource-based livelihoods such as agriculture [6,7]. The agriculture of the deltaic region will be more affected because of land submergence, salinization of soil and fresh groundwater, and losses caused by permanent coastal erosion with consequences of farm production, livelihood diversification, household well-being, and food security [8]. 

Vulnerability to climate change is driven by biophysical and socio-economic factors that intensify the susceptibility of a community to the impact of climatic stressors [9,10]. Many of the studies focusing on climate change risk and adaptation considered vulnerability assessment, and a majority of them interpreted vulnerability as a pre-existing condition while some others considered as an outcome [11]. Over the decades, the concept of vulnerability has evolved from many research disciplines: human ecology, political ecology, physical science, and spatial analysis [12,13], and these diverse approaches have resulted in different interpretations of the term ‘vulnerability’. However, much of the literature on vulnerability has applied the IPCC definition: “the degree to which a system is susceptible to and unable to cope with, adverse effects of climate change, including climate variability and extremes. The vulnerability is a function of the characters, magnitude, and rate of climate change and variation to which a system is exposed, its sensitivity, and its adaptive capacity” [14]. In general, there are two approaches to measuring vulnerability, i.e., vulnerability variable assessments, and the indicator approach [15]. Currently, indicator-based assessments are one of the most common ways to quantify climate change-led vulnerability [16], but they have faced widespread criticism for not being able to capture the complexity of vulnerable systems [17], and their lack of uniformity, particularly in the process of selecting the indicators, scale of measurement, weighting, variable transformation, and aggregation [18,19,20,21,22]; indeed, this remains a challenging task in constructing a robust vulnerability index [23]. However, there is consensus among the scientific community that indicator-based assessments can serve as a decent starting point for the analysis and discussion of vulnerability [13], especially when visualization techniques are applied [23]. 

Since agriculture is one of the sectors most vulnerable to climate change, efforts are being attempted to outline and compare different levels of agricultural vulnerability by generating composite indices based on sets of indicators by reflecting multiple dimensions of the vulnerability concept, capturing the exposure, sensitivity, and adaptive capacity of agroecological systems [16]. In fact, numerous studies [15,23,24,25,26,27,28,29] in the literature have been conducted at multiple scales and have covered different sectors of agriculture, including crops, fisheries, forestry, and livestock. The primary focus of these studies was to “help policymakers identify ‘hotspots’ in allocating adaptation resources, communicate climate risks to the public, monitor the effects of adaptation measures, and understand the weakness in the socio-ecological system that leads to vulnerability” [30]. However, most of these studies showed ‘snapshot’ views of agricultural systems’ vulnerability by incorporating some commonly used indicators [23], and little research has focused on agricultural practices in small densely populated countries whose economies are predominantly based on subsistence farming; such countries differ widely from countries with large but low-density populations, where commercial farming prevails and farms are less diversified. 

Bangladesh is an agro-economy-based developing country [31] where agricultural livelihoods, particularly in the coastal region, are becoming more vulnerable to increased intensity of climatic variability and extreme weather events (floods, droughts, storm surges and cyclones), along with environmental degradation (salinization, inundation and soil erosion) over different time horizons [8,32]. The coastal communities, in particular, have a high exposure to these stressors [33,34,35,36,37,38]. For example, the last two mega-cyclone events—Sidr and Aila—occurred in 2007 and 2009, causing a large number of human casualties; losses to the economy, agriculture and infrastructure; and imbalances in ecological processes; and ruining the livelihoods of millions of people, eventually instigating mass migration from the southwestern coastal region to other areas of the country [35,38,39]. Meanwhile, about 63% of the cultivable area in the coastal zone is affected by various degrees of soil salinity and predicted that a one-meter rise of sea level will place almost 20% of the land area of Bangladesh under the sea and cause 20–30 million people to be displaced from the coastal zone [36]. The production of oilseed, jute, and sugarcane has already been discontinued as a result of salinization, triggering land-use change towards saline aquacultures like shrimp or rice-shrimp systems that adversely affect the environment [8]. Hence, adapting to an unprecedented rate of climate change is becoming the biggest challenge, prompting an urgent need for broader adaptation options [40] for safeguarding the jeopardized agricultural livelihoods of coastal people and anticipation of even greater climatic variation in the future [34,41,42]. Assessing agricultural livelihood vulnerability to climate change is therefore imperative to formulate and implement targeted adaptation strategies and setting priority areas for investment in the agricultural sector [43].

Studies focusing on vulnerability assessment have so far not been comprehensive or appropriately delineated, especially in the coastal region of Bangladesh. Although a large number of vulnerability studies [27,34,35,38,42,44,45,46,47,48,49,50,51,52,53,54,55] are being conducted, almost none of the previous studies addressed integrated agriculture (i.e., crops, fisheries, and livestock) or took into account the connections between different spatial scales, addressed multiple hazards, or led to the formulation of intervention planning to enhance adaptation capacity. These latter two issues comprise a global research gap [56]. Notably, vulnerability assessment of the farming sector was restricted to a localized small area [46,55] and specialized fields like fisheries [27]. Hence, the agricultural vulnerability in coastal Bangladesh needs to be assessed by capturing fisheries and livestock along with the crop sector because, in reality, the elements of this tri-economy are invariably mutually dependent and most productive agriculture in the Asian region is located around the river—floodplains, swamp, lakes, and other water reservoirs [57]. So these three major farming enterprises deserve to be interconnected [58] to assess climate change vulnerability for a country like Bangladesh, which is driven by an agro-based economy. Furthermore, vulnerability assessment in coastal Bangladesh should be conducted to the administrative unit (district) at which government agencies allocate resources, which is one mechanism to help ensure that output from vulnerability assessments can be integrated into strategic government plans. Such an assessment at the district level could empower decision-makers and other non-governmental bodies to effectively direct the adaptation investment [59].

Therefore, this study aims to (i) develop a framework for assessing agricultural livelihood vulnerability to climate change and apply it to the coastal region of Bangladesh; (ii) map out the hot spots of vulnerability distribution; (iii) identify critical factors of spatially heterogeneous vulnerability; and (iv) support intervention planning for climate change adaptation. The agricultural livelihood vulnerability index (ALVI) model, built upon the integration of socioeconomic, agro-ecological, and biophysical variables (64 in all) in the vulnerability concept is a novel approach because it adds some new robust indicators such as a salinity severity index, arsenic problems, and the use of different agro-technologies. 

## 2. The Agricultural Livelihood Vulnerability Index: Conceptual Framework

The vulnerability assessment of our study takes as its starting point the IPCC typology, which describes climate change-led vulnerability as a function of exposure, sensitivity, and adaptive capacity [14]. The reasons for adopting the E-S-A framework are threefold: first, it accumulates the main elements of socioeconomic and ecological systems at multiple scales; secondly, it accentuates adaptive capacity, which determines the level of vulnerability to a greater extent; and finally, it affords an integrated assessment by capturing a diverse set of layers and suitable indicators. 

**Figure 1:**
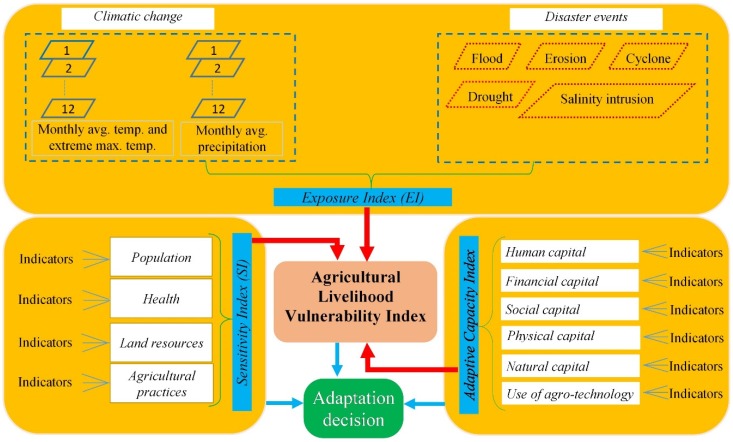
Framework of vulnerability assessment.

Agricultural livelihood refers to “individuals or communities whose livelihoods depend on crops, livestock, fish, trees, and other renewable resources” [1]. In this study, agricultural livelihood vulnerability is defined as the degree to which the agricultural sector and dependent economic endeavors are unable to recover from or adapt to the adverse impacts of climatic variability and disasters affecting farming practices. Therefore, livelihoods within an agricultural community will be more vulnerable if the community is highly exposed to the effects of climatic variability and extreme events such as erratic rainfall and cyclones; shows great sensitivity to crop, fisheries and livestock production along with demographic pressures and health susceptibilities; and at the same time has inadequate adaptive capacity such as livelihood capital and adaptive agro-technology (Figure 1). 

## 3. Methods 

### 3.1. Profile of the Case Study Area 

**Figure 2:**
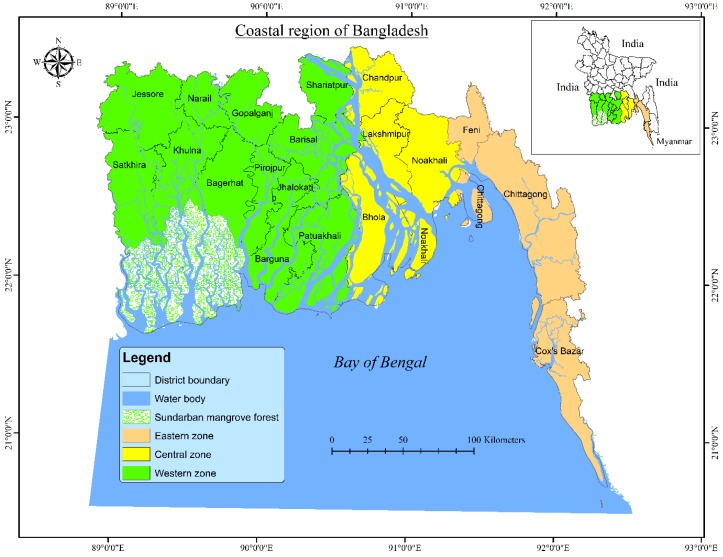
Case study area map showing the entire coastal region of Bangladesh with the different coastal zones and districts.

The present study covered the entire coastal region of Bangladesh lying between 20°6′ N to 23°5′ N latitude and 88°5′ E to 92°6′ E longitude (Figure 2). It comprises 32% (47,201 km^2^) of the total geographical land area of Bangladesh [36,38] and is home to about 40 million people in 19 administrative districts, where almost 80% of people are living in rural areas, and more than 70% of them are involved with agriculture-related activities [60]. This region is unique from ecological and economical points of view, containing a world heritage Sundarban mangrove forest (6017 sq. km), the world’s longest natural sea beach (120 km), coral islands, mountains, tidal estuaries, renewable and non-renewable energy resources, productive agricultural lands and marine resources [55,61]. The entire coastal region of Bangladesh is divided into three distinct zones: east coast, central coast, and west coast (Figure 2). The eastern coast is characterized by higher elevations and stable landmasses, and has experienced massive land-use changes; whereas the central coast is characterized by the Ganges-Brahmaputra floodplain, an active delta at a lower elevation, and has suffered massive erosion and accretion of sediments from the strong currents and tides. On the other hand, the west coast is a mature delta with large saline areas; it harbors a mangrove forest, yet experiences substantial anthropogenic pressures [62]. The entire coastal region; however, is characterized by low-lying topography: 62% of the land is below 3 m elevation, and about 86% is below 5 m above average sea level [55,61].

### 3.2. Indicator Selection, Data Collection and Transformation to Spatial Scale

In general, three diverse research streams are underlined in the literature regarding agricultural vulnerability to climate change: biophysical, agro-ecological, and socioeconomic aspects [24]. The biophysical aspects consider agricultural systems’ exposure to climate change and variability by incorporating indicators like precipitation variability, the occurrence of flood, drought, and environmental degradation [28]. The agro-ecological dimensions are represented by the sensitivity of farmland and production to climate shocks [26,28]. The socioeconomic aspect is reflected by analyzing climate change’s impact on agricultural productivity and farm income [24] as well as social vulnerability, which primarily incorporates indicators relating to vulnerable social groups and their capacity to adapt to climate change [23,24,28,29]. The indicator method of this study consists of 64 indicators (see details in Table 1) reflecting socioeconomic, agro-ecological, and biophysical variables.

The time-series data (1964–2013) on maximum and minimum temperature, and rainfall from 19 weather stations spread over the entire coastal region, were obtained from the Bangladesh Meteorological Division (BMD), Agargaon, Dhaka. The historical data on frequency and intensity of floods and cyclones were collected from the Bangladesh Bureau of Statistics (BBS) report, and the hazard score was computed following the methodology of Barua et al. (2016) [63]. The district-level average drought intensity score was obtained from Alamgir et al. (2019) [64], calculated based on the time series precipitation data during 1994–2013 for the Kharif season. 

The district-level data on area and intensity of salinity were collected from the Soil Resources Development Institute (SRDI). The salinity severity index was calculated using Equation (1):

(1) $$\mathrm{Salinity}\;\mathrm{Severity}\;\mathrm{Index}\;\left(\mathrm{SSI}\right)=\frac{\sum_{i=0}^{5}S_{i\;}A_{i}}{\sum_{i=0}^{5}A_{i}}$$

where S_i_ represents the salinity class; A_i_ represents the % area under the i^th^ salinity class, i.e., i = 0, 1, 2, 3, 4, 5. S_0_ = no salinity or < 2 ds/m; S_1_ = 2–4 ds/m; S_2_ = 4–8 ds/m; S_3_ = 8–12 ds/m; S_4_ = 12–16 ds/m; S_5_ = above 16 ds/m.

Seven Landsat TM and OLI-TIRS scenes (30×30-m resolution) covering the entire coastal region of Bangladesh for the years 1998 and 2018 were collected from the LSDS Science Research and Development (LSRD) database of the United States Geological Survey (USGS) (https://espa.cr.usgs.gov/) (free of cost); these were used to calculate the land use/land cover (LULC) changes (Figure S1 and Table S1). The rate of riverbank erosion (km/year) was estimated based on the dynamics of LULC change [65], and the land-use intensity (LUI) was estimated by considering the results of LULC following Huang et al. (2012) [66].

The land degradation index was constructed based on key informants’ perception analysis [29]. Components of land degradation assessment in this study included anthropogenic activities, i.e., agricultural mismanagement, overgrazing, fuel-wood consumption, deforestation, industry, and urbanization. 

The cross-sectional data on land resources—i.e., soil organic matter, soil phosphorous; agricultural practices, livelihood capital, and agro-technology use at the district level—were compiled from the districts’ statistical reports (Jila Batayan) and the Agricultural and Fisheries statistical yearbook of Bangladesh published by BBS. 

### 3.3. Index Formation and Spatial Mapping

Since the collected data had different ranges and scales, they were normalized to rescale within a dimensionless range (0–1) for ensuring uniformity and comparability of the indicators. We used inverse values of some sensitivity and adaptive capacity indicators to ensure that the increase in value always represented an increase in sensitivity and adaptive capacity [57].

The relative weights of the 64 indicators (see Table 1) under three major components were estimated using the analytic hierarchy process (AHP) [67]. The consistency ratio (CR) in AHP ranged between 0.1 and 8.8%, which was satisfactory [68] (see details of the AHP approach in the supplementary materials and Tables S2 and S3). Since different variables affect vulnerability unevenly, the equal weight method was not used. Moreover, statistical methods are often criticized for ignoring local knowledge and traditional values [15]. 

This study applied a weighted sum of sub-indices [18,57] technique for aggregation purposes. Therefore, exposure, sensitivity, and the adaptive capacity index were assumed as the linear sum of their indicators, and measured according to Equation (2):

(2) $$\mathrm{EI},\;\mathrm{SI},\;\mathrm{ACI}=\;\sum_{i=1}^{m}W_{i}Y_{i}$$

where EI, SI, and ACI represent the values of the exposure index, sensitivity index and adaptive capacity index, respectively; W_i_ represents the weight of the i^th^ indicator (i = 1, 2, …, m); and Y_i_ represents the normalized value of the i^th^ indicator. A similar technique was applied for analyzing the index of the 12 (twelve) sub-components. 

In this study, agricultural livelihood vulnerability to climate change was determined by subtracting the adaptive capacity index from the arithmetic sum of the exposure index and sensitivity index [28,69] following Equation (3):

Vulnerability = {(Exposure + Sensitivity) − Adaptive capacity}

ALVI = (EI + SI − ACI)(3)

Further normalization of calculated EI, SI, ACI and ALVI indices provided us with index values between 0 and 1, where a value closer to 0 means a lower level of relationship to EI, SI, ACI or ALVI, and a value closer to 1 means a higher level [69]. The categorization of EI, SI, ACI, and ALVI into five classes of attributes (very low, low, moderate, high, and very high) at the spatial scale was accomplished by employing the equal interval approach under an ArcGIS environment [57,69]. Furthermore, the coefficient of correlation was run to find the relationships among EI, SI, ACI, and ALVI [26,56]. 

### 3.4. Hot Spot Analysis

We used an Exploratory Spatial Data Analysis (ESDA) technique—spatial autocorrelation—to analyze the distribution characteristics of ALVI across the coastal districts of Bangladesh. The spatial autocorrelation analysis was performed through GeoDa-1.12.1.161 software following the methodology of Jha and Haripriya (2019) [69]. In spatial autocorrelation, Moran’s I designates a value score range from +1 to −1, which indicates the spatial pattern between the neighboring regions and observations [69]. A Moran’s I score that is close to +1 shows a strong similarity pattern between the high and low values, whereas −1 reflects a strong dissimilarity pattern indicating a varied pattern of high and low values. On the other hand, LISA (Local Indicators of Spatial Association) identifies four types of spatial clusters—HH (high-high), HL (high-low), LH (low-high), and LL (low-low)—at the local level. An HH value indicates a region of high ALVI values surrounded by other regions of high ALVI values, and is referred to as ‘hot spot’; whereas LL values represent a region with low ALVI scores bounded by less vulnerable regions and is referred to as ‘cold spot’. The HL and LH areas are those with extreme values, reflecting a negative spatial autocorrelation, and are referred to as ‘spatial outliers’. 

### 3.5. Development of Intervention Plan

Since uncertainty is interlinked with climate change, exposure is beyond the reach of policy interventions. However, adopting suitable policy interventions for reducing sensitivity and enhancing adaptive capacity could reduce vulnerability [56]. To this end, district-wise sensitivity and adaptive capacity index values were plotted on the X and Y axes, respectively, of a scatter diagram, to develop a decision matrix that could help identify socioeconomically vulnerable areas so that effective interventions could be taken, on a priority basis. Districts in the quadrants with SI scores ≤ 0.50 and ACI scores > 0.5 were treated as low vulnerability areas, whereas highly vulnerable districts were recognized when SI score > 0.5 ≤ ACI score. Furthermore, the normalized relative values (rescaled) of the indicators under sensitivity and adaptive capacity components were plotted on circumplex charts, to identify the drivers (sensitivity indicator value > 0.5 ≥ adaptive capacity indicator value) and buffers (sensitivity indicator value ≤ 0.5 < adaptive capacity indicator value) by using the mean value (0.5) as a threshold boundary.

## 4. Results and Analysis

### 4.1. Exposure Dimension 

**Figure 3:**
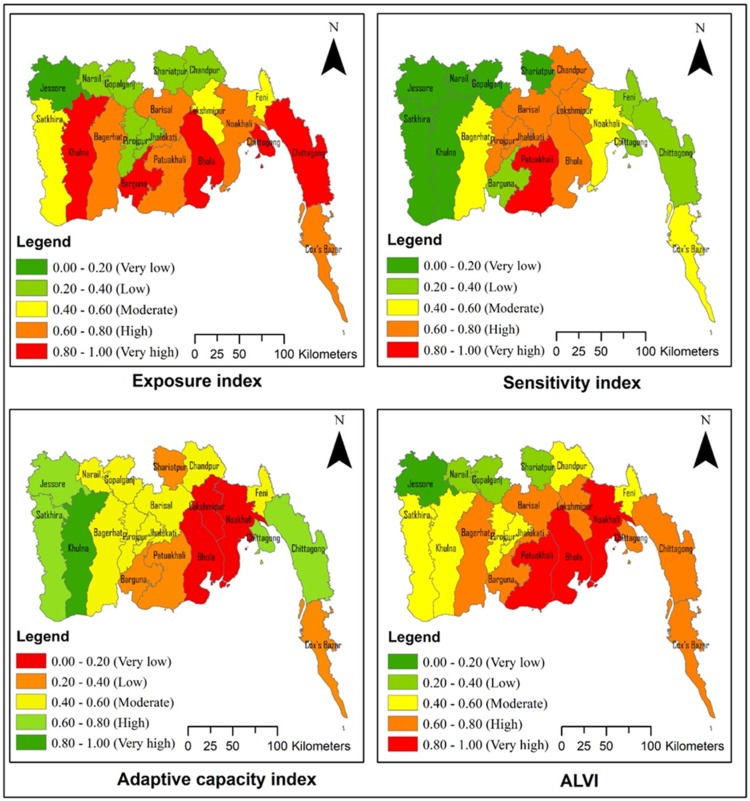
Mapping of spatial variation of exposure index, sensitivity index, adaptive capacity index, and agricultural livelihood vulnerability index, across the coastal districts.

Aggregation of the weighted value of indicators under the exposure dimension reveals (Figure 3, Table S4) that four (Bhola, Khulna, Barguna, and Chittagong) out of the 19 districts had very high levels of exposure, with index scores of 0.560–0.651. However, the Bhola district is the only island district, and it had the top-ranked exposure index value (0.651), which corresponded to its higher exposure to erosion, flood, cyclone, and drought. The Barisal, Patuakhali, Bagerhat, Noakhali, and Cox’sbazar districts had exposure index scores between 0.482–0.566 and were grouped into districts with high exposure to climate disasters. The aggregated land area of the high to very high exposure districts accounted for 65.87% of the total study area, indicating that two-thirds of the coastal region is highly exposed to climate disaster. Approximately 23.33 million people (60.59% of the total population) are living in the districts located in these highly exposed areas. On the other hand, seven districts (Jessore, Jhalokati, Narail, Gopalganj, Pirojpur, Shariatpur and Chandpur), mainly distributed along the interior coast and had low to the very low exposure index values, and their aggregated land area accounted for 20.87% of the total study area. 

### 4.2. Sensitivity Dimension 

Agricultural susceptibility indicates the probability of the agricultural farming sector’s being affected by the impacts of climate change. Findings (Figure S2 and Table S5) revealed that ten (Barisal, Chandpur, Satkhira, Khulna, Bagerhat, Patuakhali, Bhola, Lakshmipur, Pirojpur, and Jhalokati) out of the nineteen districts had high to very high levels of agricultural susceptibility, and their aggregated land area accounted for 56.52% of the total study area. The Patuakhali district had the highest susceptibility score (0.315) of agricultural practices sub-dimension, which corresponded to a significant proportion of marginalized farm holdings (34.24%), crop area (60.43%), rain-fed agricultural land (95.54%), with a higher gross agricultural production (1.15), and low yield (1.99 ton/ha) of rice. 

Combining the indicators under the sensitivity component reveals (Figure 3 and Table S4) that the Patuakhali district had the highest sensitivity score (0.655) and was categorized as having very high sensitivity to climate change. The districts of Chandpur, Bhola, Noakhali, Lakshmipur, Jhalokati, and Pirojpur were grouped into the category of high sensitivity, with index scores of 0.542–0.604. The aggregated land area of the high to very high sensitivity districts accounted for 41.25% of the total study area. Hence, the agricultural livelihoods of 14.68 million people (38.13%) are highly sensitive to climate change, and this area corresponded to the dominance of crop-based (58.16%) and rain-fed (76.34%) farming; low crop diversity (1.83) and productivity (rice ~ 2.42 ton/ha); and low soil phosphorus (12.38 µg/gm) and organic matter (2.09%) content. Moreover, these highly sensitive districts accommodate the highest proportion of the rural (88.20%), marginalized (37.87%) and economically poor (23.05%) population with unhygienic sanitation conditions (45.34%), distant water sources (21.58%), and high infant mortality rates (47.50%). 

### 4.3. Adaptive Capacity Dimension

Findings suggests (Figure 3 and Table S4) that the Khulna district alone fell under the category of very high adaptive capacity, with an index value 0.562, corresponding to its higher proportion of employed population (35%), female workforce (6.30%), income diversification (IDI = 5347.11), medical density (15.39/100,000 people), structurally sound houses (41.30%), dams (25.06%), natural forests (46.21%) and lower fertilizer application (0.11 ton/ha). The districts Jessore, Satkhira, and Chittagong had index scores between 0.460–0.510 and were categorized under the high adaptive capacity group. On the other hand, seven (Shariatpur, Barguna, Patuakhali, Bhola, Lakshmipur, Noakhali and Cox’sbazar) out of the nineteen districts had low to very low adaptive capacity, with index values ranging from 0.306 to 0.406. The aggregated land area of the low to very low adaptive capacity districts accounted for 37% of the total study area, which was mainly distributed in the high-exposure and -sensitivity zone and signifies their vulnerability to future climate change impacts. Critical factors of low adaptive capacity corresponded to the districts’ literacy rate (50.14%), structurally sound houses (12.60%), dependence on agriculture (55.94%), and adoption of agricultural technologies such as improved crop variety (55.75%), irrigation pumps (27.38%), and harvesters (9.82%). 

### 4.4. Agricultural Livelihood Vulnerability Index

The ALVI score at the district level ranged from 0.177 to 0.882. Results (Figure 3 and Table S4) revealed that three (Bhola, Patuakhali, and Noakhali) out of the nineteen districts had very high levels of vulnerability, with ALVI scores of 0.759–0.882. The Bhola district—the only island district—ranked first in ALVI, which corresponds to very high exposure (0.651), moderate sensitivity (0.519), and very low adaptive capacity (0.342). The Barisal, Bagerhat, Barguna, Lakshmipur, Chittagong, and Cox’sbazar districts had ALVI scores of 0.605–0.716 and were grouped under the high vulnerability category. The aggregated land area of the high to very high vulnerability districts accounted for 59.60% of the total study area, signifying that 22.75 million people (59.07%) are highly vulnerable to climate change. The moderate vulnerability districts, with ALVI scores of 0.450–0.543. included Satkhira, Khulna, Pirojpur, Jhalokati, Feni, and Chandpur. Only four districts (Jessore, Narail, Gopalganj, and Shariatpur) had low to very low ALVI, and all of these districts are situated in the interior part of the western coastal region. This finding is in accord with Quader et al. (2017) [42], who concluded that some island areas of the eastern zone are very susceptible to cyclone hazards. A comparatively high vulnerability to salinity intrusion was reported in the southwestern region [51], while fisherman’s livelihoods were more vulnerable to climate change in the Patuakhali district than in Cox’sbazar [27]. A higher level of vulnerability and livelihood risk existed in the more cyclone-affected areas [42], while saline-prone areas were found to be more vulnerable than flood- and drought-prone areas [52].

Correlation analysis (Table S6) revealed that both the EI and the SI showed very highly significant positive relationships with ALVI (r_ALVI vs.EI_ = 0.833 **, *p* < 0.01; r_ALVI vs.SI_ = 0.620 **, *p* < 0.01). However, ACI showed a very highly significant negative correlation with ALVI (r_ALVI vs.ACI_ = −0.524 *, *p* < 0.05). Hence it could be expected that the exposure, sensitivity, and adaptive capacity dimensions had almost equal influences in determining the agricultural livelihood vulnerability of the coastal communities to climate disasters. An almost identical phenomenon was observed in some other Asian countries, where sensitivity [26,56] and adaptive capacity [25,26,56,57,70] predominantly influence vulnerability to climatic hazards. Furthermore, a very weak correlation (r_EI vs.SI_ = 0.240; r_EI vs.ACI_ = −0.093, r_SI vs.ACI_ = −0.284, *p* > 0.05;) was found among the major components, suggesting that these three components occur independently, a result that also supports the findings of Li et al. (2015) [26], who assessed agricultural vulnerability in China, and Krishnan et al. (2018) [56], who assessed coastal vulnerability due to climate change in India. 

### 4.5. Hot Spots and Factors of Spatially Heterogeneous Vulnerability

**Figure 4:**
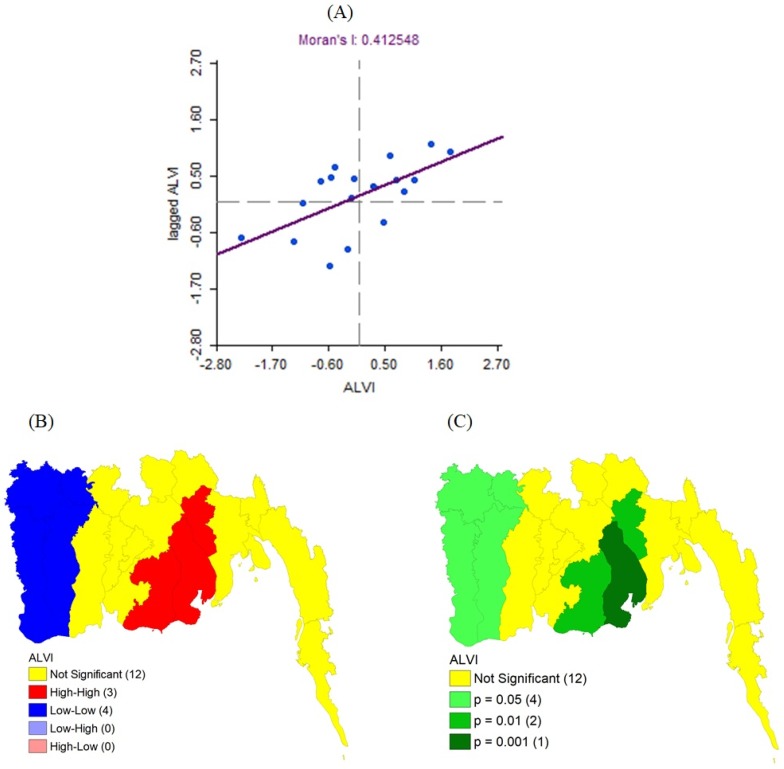
Spatial analysis showing the pattern of vulnerability distribution across the study area: (**A**) Moran scatter plot of the ALVI values; (**B**) LISA cluster map; (**C**) LISA significance map. The red and blue color areas in map (**B**) indicate ‘hot spots’ and ‘cold spots’ of vulnerability distribution, respectively.

The Moran scattered plot (Figure 4A) provided a relatively high Moran’s I score (0.4125) of the ALVI, indicating that the distribution pattern of the ALVI in the study area exhibited evident clustering, displaying a strong positive correlation. According to Figure 4B, only two types of significant autocorrelations could be found in the study area: High-High (HH), and Low-Low (LL). Consequently, we did not find any High-Low (HL) or Low-High (LH) spatial outliers, and the remaining groups were found to be insignificant (Figure 4B). Figure 4B shows that the values of the HH cluster were mainly concentrated along the mouth of the Meghna estuaries and distributed in the three districts Bhola, Patuakhali, and Lakshmipur, which were categorized as highly (Lakshmipur) to very highly (Bhola and Patuakhali) vulnerable districts (Figure 3). On the other hand, LL clusters were concentrated in the southwestern coastal region and distributed in the four districts Satkhira, Khulna, Jessore, and Narail. The LISA significance map (Figure 4C) shows that the significance of ALVI in HH and LL was 0.001 to 0.05, which indicates a strong positive correlation, and signifies that the ALVI values of these districts are positively related to the ALVI of neighboring districts. 

In this study, we disaggregated indicator values across vulnerability classes in the ANOVA test to discover the factors of spatial differences in the agricultural livelihood vulnerabilities, among the coastal districts. As was revealed in the ANOVA test results (Table 2), there are significant spatial differences in riverbank erosion, cyclone hazard, and drought intensity with regards to exposure components which contributed to describing the differential level of vulnerability among the coastal districts. Furthermore, spatial differences of infant mortality rate, unhygienic sanitation conditions, land degradation, soil phosphorus, rain-fed agricultural land, and crop productivity that corresponded different levels of agricultural sensitivity to climate change and described heterogeneous vulnerability across the coastal districts. On the other hand, spatial variation of structurally sound houses, emergency shelters, open waterbody, improved crop variety, and use of pesticides and irrigation pumps have influenced adaptive capacity and resulted differential level of vulnerability to climate change. In other words, these are the factors with regards to exposure, sensitivity, and adaptive capacity that act as key factors of heterogeneous vulnerability among the coastal districts. 

### 4.6. District-Level Intervention Planning 

**Figure 5:**
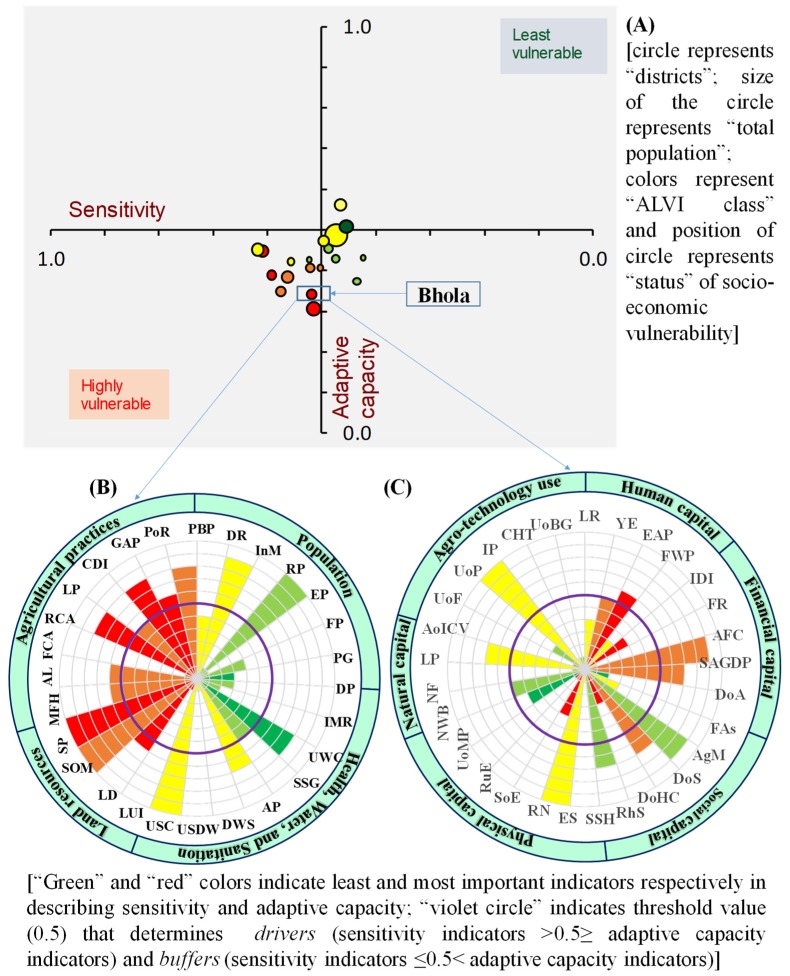
Decision matrix for entire coastal region incorporating 19 coastal districts by plotting sensitivity indices against adaptive capacity indices (**A**) and visualization of drivers and buffers of both Sensitivity and adaptive capacity components of a representative district on the circumplex charts (**B**,**C**), respectively, for intervention planning.

As shown in Figure 5A, all the districts having high to very high ALVI were placed in the third quadrant (highly vulnerable), indicating that those districts are socioeconomically more vulnerable and should be prioritized for intervention planning. 

The circumplex chart (Figure 5B) for a sample district, Bhola (similar results for other districts are compiled in Figures S3 and S4), shows that dependency ratio (DR), rural population (RP), underweight children (UWC), unhygienic sanitation conditions (USC), soil organic matter (SOM), soil phosphorus (SP), rain-fed agricultural land (RAL), crop diversity index (CDI), and productivity of rice (PoR) were the key drivers contributing to high sensitivity in the Bhola district, implying that vulnerability was structured to some extent, due to a higher rate of rural smallholder farm households and their dependency on agri-based livelihoods, with low diversity and productivity of the land. On the other hand, the primary drivers that lowered the adaptive capacity of the Bhola district were found to be literacy rate (LR), female work participation (FWP), income diversity index (IDI), foreign remittances (FM), dependency on agriculture (DA), farmers’ associations (FAs), density of healthcare facilities (DoHC), structurally sound houses (SSH), road network (RN), share of embankments (SoE), rural electrification (RE), natural forest area (NFA), adoption of improved crop varieties (AoICV), use of fertilizer (UoF), irrigation pumps (IP), crop harvesters/threshers (CHT), and use of biogas (UoB) (Figure 5C). Diversifying agricultural systems, implementing irrigation facilities and technology-based farming practices, education, sanitation, income diversification by non-agricultural industries and infrastructural development—especially disaster-resistant houses, electricity, and roads—are therefore some of the prioritized actions to be considered to reduce the sensitivity and enhance the adaptive capacity of Bhola.

## 5. Discussion

### 5.1. Implication of Relative Spatial Vulnerability among Districts 

At a glance, the vulnerability map suggests that all the top-ranked vulnerable districts are distributed at the mouth of the famous deltaic Meghna estuaries and the Ganges-Brahmaputra coastal plain adjacent to the south-central coastline. This finding partially supports the previous vulnerability assessments [27,51], while disagreeing with regard to agricultural vulnerability assessment of Uddin et al. (2019) [48]. Uddin et al. (2019) [48] considered that only two variables—rice production and irrigation pump use—reflected agricultural vulnerability, and applied the PCA (principal component analysis) method. The ALVI approach, however, includes more indicators to represent the diversified agriculture of coastal Bangladesh, and applies unequal weighting of indicators by the experts’ judgment, providing more accurate results [15,18,21,56,71]. The most vulnerable region was also found to be the most exposed to climatic variability and disasters, compared to other districts, a result in agreement with the hazard map of Bangladesh developed by CEGIS [42] and some other literature [48,63].

A closer inspection of the vulnerability map; however, reveals that the top two most vulnerable districts (Bhola and Patuakhali) possessed higher exposures and sensitivities and lower adaptive capacity, and are in a hot-spot zone. The higher level of adaptive capacity and low sensitivity of the Khulna district, on the other hand, ameliorated its high exposure to climate disaster. In contrast, the Chittagong district, despite its high level of adaptive capacity and low sensitivity, was influenced by a very high level of exposure and pushed the district to higher vulnerability. On the other hand, exceptionally low adaptive capacity, coupled with high exposure and moderate sensitivity, pushed Noakhali towards being a very highly vulnerable district. In general, districts in the interior coast had less exposure, sensitivity, and overall vulnerability to climate change. 

A crucial finding was a very high level of vulnerability in the Patuakhali district. The district is categorized as highly prone to climate disasters, especially cyclones, sea level rise [27], and low tendency to adopt innovative agricultural technologies [46]. This study supports previous findings regarding farm technology use capacity and high exposure to cyclones. However, the Patuakhali district is less exposed to precipitation variability, flood hazards, and extreme temperature events, and, combined with a relatively lower level of literacy, industrial workers, structurally sound houses, road density, electricity, agricultural diversity index, crop productivity, and cropping intensity, and a higher level of rain-fed cropland, dependence on agricultural production, and unhygienic sanitation conditions triggered the district’s high vulnerability. 

### 5.2. Benefits of the ALVI Approach

The ALVI can be useful in assessing the impact and effectiveness of a program or policy by producing a model index value of contributing indicators and thereby yielding an updated ALVI score. For example, if the purpose of a disaster risk reduction program is to minimize crop loss, the length of dams being constructed in a geographical area over a stipulated period could be incorporated, and new ALVI scores calculated. The new ALVI could then be contrasted with the baseline ALVI to estimate the intervention’s effects on the district’s agricultural vulnerability to climate change. 

The purpose of developing the ALVI model was to present an integrated assessment tool that can cover the multidimensionality of the aspect being assessed with minimal data limitations. It allows us to improve on the strategies utilized by prior studies, by covering all aspects of the coastal locale—rural and urban, island and mainland—and carefully utilizes a combination of census, survey, meteorological, and spatial data to overcome data limitations as far as possible. By using data from multiple years, the changes in ALVI score over a specific period can be measured. For instance, we have used most of the socioeconomic data for the year 2011, which could be used as baseline data to be compared to data on the same variables, which will be readily available in the year 2021 (the target year for the Bangladesh Government’s statistical division) and will allow the change in ALVI to be calculated over a 10-year period. 

The ALVI approach uses a spatial autocorrelation technique to discover the hot spots of vulnerability distribution (Figure 4) and develop a strategy to find the drivers of vulnerability (Figure 5). The results can be used to develop an intervention plan, to help policymakers target specific geographic locations where they can take intervention measures, easily selecting an area on a priority basis. Spatial autocorrelation also helps find the cold spots—the areas where vulnerability is very low, enabling progressive changes in the more vulnerable areas by copying the successful adaptation strategies of these cold spots. 

The proposed ALVI model can also be used as a generalized operational method in other geographical areas, to accumulate multi-dimensional spatial information that can be used to identify vulnerability and assist policy-makers in supporting climate-change adaptation. However, the selection of variables requires a careful and methodical assessment, as climate change-induced vulnerability is a complex phenomenon and is strongly correlated with local socioeconomic, ecological and biophysical conditions. Furthermore, a decent data management strategy at an appropriate spatial scale is needed for an accurate reflection of agricultural sector vulnerability through ALVI. 

### 5.3. Limitations of the Study and the ALVI Approach

Since vulnerability is a multidimensional concept and not directly measurable, it is associated with a high level of uncertainty in the indicator selection, measurement, and classification processes [71]. First, it was challenging to select the specific indicators for crops, fisheries, and livestock, because different crops, fish species, and livestock varieties are found in different districts, and uniform indicators had to be adopted across the districts. 

Secondly, the trend of agricultural production and the extent of technology use were considered in this study, rather than simply using an existing crop model to predict the future scenario of agricultural output. However, linking a crop model in multi-indicator approaches could be more useful for estimating the sensitivity and adaptive capacity of agriculture [25,72]. 

Thirdly, it was challenging to find district-level historical data on climate disasters and, most importantly, for the same period of time. Moreover, a few districts did not have any weather stations, and for those districts, data from the nearest weather station were used. Therefore, there is room for reducing the uncertainty of vulnerability assessment, if more specific data become available for the same period. 

Finally, we classified the vulnerability of a coastal district based on a beta distribution of vulnerability index scores. However, it is expected that the classification of vulnerability may not prevail over the long term because an improvement in adaptive capacity may moderate climate change impacts in the future [14]. 

## 6. Conclusions

Understanding and prioritizing which areas and communities, at sub-national scales, are most vulnerable to climate change has become a growing concern in policy circles in order to develop a sustainable adaptation plan. We developed an ALVI method for assessing the relative vulnerability of coastal agricultural livelihoods to climate change impacts with regards to the spatial variations of climatic change, disaster events, demography, health, land resources, agricultural practices, livelihood capitals, and agro-technology use. The proposed framework could help in identifying the most vulnerable geographic units and their hot spots for prioritized attention. It can also help distinguish the causal factors to existing vulnerability, referred to in this study as ‘drivers’ and ‘buffers’, with the former being identified as aspects for prioritized investment to support adaptation intervention. 

The investigation, using an ALVI framework in coastal Bangladesh—a representative area of integrated and subsistence agricultural farming, which is particularly threatened by climate change—revealed that the agricultural livelihoods of 22.75 million people in 9 administrative districts are predominantly vulnerable to climate change, most notably (i) the Bhola district, due to low soil phosphorus and organic matter content, a larger area of rain-fed cropland, low crop diversity and productivity (sensitivity); the lowest level of foreign remittances, income diversity, agro-technology use, structurally sound houses (adaptive capacity); and high levels of erosion, drought, and cyclones (exposure); and (ii) the Patuakhali district, due to salinity intrusion, drought, and cyclones (exposure); low cropping intensity, diversity and productivity, and a larger area of rain-fed cropland (sensitivity); relatively lower levels of literacy, structurally sound houses, road density, electricity, and agro-technology use (adaptive capacity). 

The hot spot of vulnerability distribution was concentrated in the rural agricultural districts (Bhola, Patuakhali, and Lakshmipur), where livelihoods are mainly dependent on crop-based farming and are continuously threatened by multiple climatic disasters such as floods, erosion, and cyclones. On the other hand, the vulnerability cold spots were distributed along the world’s largest mangrove forest, the Sundarbans, which offers numerous livelihood opportunities and reduces the vulnerability of surrounding districts (Satkhira, Khulna, Jessore, and Narail) by providing an ecological buffer against climatic disasters. Furthermore, the spatially heterogeneous vulnerability among the coastal districts was influenced by the indicators of exposure (rate of erosion, cyclones, and drought); sensitivity (infant mortality rate, distance to water source, unhygienic sanitation conditions, land degradation, soil phosphorus, rain-fed agricultural land, productivity of rice); and adaptive capacity (structurally sound housing and density of emergency shelters, open waterbody, adoption of improved crop varieties, pesticides, and irrigation facilities). 

The proposed assessment method provides a concrete example of a set of potential adaptation measures for specific geographical units that will assist policymakers in prioritizing investments for intervention. For example, diversification of agricultural systems by allowing water-intensive crops; adoption of farming technology (crop variety, harvester use, irrigation pumps); construction of dams and roads, and enhancing the plantation mangrove forest program, are some of the potential adaptation options for the most vulnerable district, Bhola. These measures could reduce the sensitivity and modify the agricultural system’s exposure to stressors such as flood, erosion, drought, and cyclones. Subsequently, findings of this study may accelerate the shift of adaptation efforts to areas with greater exposure, increased sensitivity, or lower adaptive capacity. 

## Figures and Tables

**Table 1:** Vulnerability components, sub-components, indicators and their functional relationships with major components and data sources, and final relative weight of indicators.

Component	Sub-Component	Indicators	Sign	Proxy	HR	Source	Time Period	Weight
Exposure	Climate	Extreme temperature	ExT	Extreme max. temp. (°C) in a 50-year return period	+	BMD	1964–2013	0.066
		Changes of temperature	CoT	Changes on average annual temperature	+	BMD	1964–2013	0.060
		Precipitation variability	PV	(Max. precipitation–min. precipitation)/avg. precipitation	+	BMD	1964–2013	0.161
	Disaster	Flood hazard	FH	Computation of flood hazard score ^a^	+	BBS	1951–2013	0.145
		Riverbank erosion	RE	Rate of riverbank erosion (km/year)	+	USGS	1998–2018	0.112
		Cyclone hazard	CH	Computation of cyclone hazard score ^a^	+	BBS	1960–2015	0.156
		Salinity intrusion	SI	Salinity severity index	+	SRDI	2010	0.185
		Drought intensity	DI	Drought intensity in Kharif season	+	*	1994–2013	0.114
Sensitivity	Population	Population below poverty level	PBP	% population below extreme poverty level	+	BBS	2011	0.014
		Dependency ratio	DR	Ratio of the population < 14 and > 65 years to that 14–65 years	+	BBS	2011	0.015
		In migration	InM	% floating people moving in from other areas	+	BBS	2011	0.014
		Rural population	RP	% population living in rural area to total population	+	BBS	2011	0.010
		Ethnic population	EP	% population living in tribal area	+	BBS	2011	0.006
		Female population	FP	% female population to total population	+	BBS	2011	0.012
		Population growth	PG	% population increased during 2001 to 2011	+	BBS	2001–2011	0.013
	Health	Disabled population	DP	% population physically disabled	+	BBS	2011	0.010
		Infant mortality rate	IMR	Infant mortality rate (no./1000 live births)	+	BBS	2011	0.010
		Underweight children	UWC	% of children under 5 years old who were underweight at birth	+	BBS	2011	0.016
		Severely stunted growth	SSG	% children under 5 years old reported as stunted growth	+	BBS	2011	0.018
		Arsenic problem	AP	% tube wells with potential threat of arsenic level > 50 mg/l	+	BBS	2011	0.022
		Distance from a water source	DWS	% households with water source greater than 200 meters away	+	BBS	2011	0.020
		Unsafe drinking water	USDW	% households drinking water from an open source	+	BBS	2011	0.116
		Un-hygienic sanitation conditions	USC	% households without hygienic sanitation facilities	+	BBS	2011	0.101
	Land resources	Land use intensity	LUI	Land use intensity	+	USGS	2018	0.045
		Land degradation	LD	Perceived land degradation index	+	Survey	2018	0.067
		Soil organic matter	SOM	Average organic matter content of soil (%)	-	SRDI	2013	0.046
		Soil phosphorus	SP	Average phosphorus content in soil (µg/gm)	-	SRDI	2013	0.040
	Agricultural practices	Marginalized farm holdings	MFH	Farm holding operating on 0.05 to 0.49 acre of land	+	BBS	2011	0.040
		Arable land	AL	% net cultivated land to total land	+	BBS	2011	0.066
		Fish-culture area	FCA	% land utilized for inland fish farming	+	BBS	2011	0.050
		Rain-fed crop area	RCA	Cropland not under irrigation facilities	+	BBS	2011	0.078
		Livestock potential	LP	Ownership of livestock (no./household)	+	BBS	2011	0.070
		Crop diversity index	CDI	Computation of CDI (Shannon diversity index) ^b^	-	BBS	2011	0.050
		Gross agri. production	GAP	Per capita annual GAP (m.ton) ^b^	+	BBS	2011	0.066
		Productivity of rice	PoR	Average yield of rice (ton/ha) in last 5 years	-	BBS	2011–2015	0.060
Adaptive capacity	Human capital	Literacy rate	LR	Literacy rate of 7+ population	+	BBS	2011	0.039
		Youth education	YE	Youth education enrollmet rate (%)	+	BBS	2011	0.042
		Economically active population	EAP	% population employed in different sectors	+	BBS	2011	0.049
		Female work participation	FWP	% female population engaged at non-home workplace	+	BBS	2011	0.030
	Financial capital	Income diversification index	IDI	Negative Herfindahl index of income diversification	+	BBS	2011	0.053
		Foreign remitter	FR	% households receiving foreign remittances	+	BBS	2011	0.030
		Access to farm credit	AFC	% households having received a loan from different sources	+	BBS	2011	0.045
		Share of agricultural GDP	SAGDP	% households with income come from agricultural sector	+	BBS	2011	0.039
		Dependence on agriculture	DoA	% households with main income dependent on agriculture	-	BBS	2011	0.015
	Social and institutional capital	Farmers associations	FAs	% population member of a cooperative society	+	BBS	2011	0.030
		Agricultural markets	AgM	No. of agricultural markets per 1000 farm households	+	BBS	2011	0.024
		Density of schools	DoS	No. of schools per 10,000 population	+	BBS	2011	0.039
		Density of healthcare facilities	DoHC	No. of healthcare facilities per 10,000 population	+	BBS	2011	0.053
		Rehabilitation support	RhS	% households receiving financial/rehabilitation support	+	BBS	2011	0.019
	Physical capital	Structurally sound houses	SSH	% houses with disaster-resistant construction	+	BBS	2011	0.036
		Emergency shelters	ES	Cyclone and flood emergency shelters (no./10,000 population)	+	BBS	2011	0.030
		Road network	RN	Road density (meter/ha)	+	BBS	2011	0.059
		Share of embankments/dams	SoE	% total embankments constructed in a district	+	BBS	2011	0.047
		Rural electrification	RuE	% rural households connected to electrical grid	+	BBS	2011	0.053
		Use of mobile phones	UoMP	% households with mobile phone	+	BBS	2011	0.018
	Natural capital	Open water bodies	NWB	% area covered by rivers and other water bodies	+	USGS	2018	0.020
		Natural forests	NF	% area under natural forests	+	BBS	2011	0.022
		Land potential	LP	Per capita land potential (total land/total population)	+	BBS	2011	0.031
	Use of agro-technology	Adoption of improved crop variety	AoICV	% rice field cultivated with HYV seed	+	BBS	2011	0.039
		Use of fertilizer	UoF	Fertilizer application rate (m.ton/ha)	-	BBS	2011	0.029
		Use of pesticide	UoP	% cropland sprayed with pesticides	-	BBS	2011	0.030
		Irrigation pump	IP	% area under irrigation facilities	+	BBS	2011	0.032
		Crop harvester/thresher	CHT	No. of harvesters/threshers per 100 farm households	+	BBS	2011	0.027
		Use of bio-gas	UoBG	% households using biogas for cooking	+	BBS	2011	0.020

HR = Hypothesized relationship between the indicator and vulnerability dimensions; TP = Time Period; BMD = Bangladesh Meteorological Department; BBS = Bangladesh Bureau of Statistics; SRDI = Soil Resources Development Institute; HYV = High Yielding Variety; ^a^ See detailed methodology in Barua et al. 2016; ^b^ See detailed methodology in supplementary information; * [64] Alamgir et al. 2019.

**Table 2:** Key factors of spatially heterogeneous vulnerability.

Dimension	Element	Indicator	F Value	Sig. Level
Exposure	Disaster events	River bank erosion	15.507	0.000
		Cyclone hazard	5.167	0.009
		Drought intensity	8.804	0.001
Sensitivity	Health	Infant mortality rate	2.548	0.086
		Distance to a water source	2.943	0.059
		Unhygienic sanitation condition	2.951	0.058
	Land resources	Land degradation	3.366	0.040
		Soil phosphorus	6.736	0.003
	Agricultural practices	Rainfed agricultural land	2.940	0.059
		Productivity of rice	3.387	0.039
Adaptive capacity	Physical capital	Structurally sound housing	4.050	0.022
		Emergency shelter	4.726	0.013
	Natural capital	Open waterbody	5.316	0.008
	Use of agro-technology	Improved crop variety	2.578	0.082
		Use of pesticide	4.219	0.019
		Irrigation pump use	2.940	0.059

## Supplementary Materials

The following are available online at https://www.mdpi.com/1660-4601/16/22/4552/s1, Table S1: Random index (RI) values, Table S2: Results of AHP analysis, Table S3: Land cover classification scheme used in this study, Table S4: Ranking of coastal districts based on the overall vulnerability index score along with exposure, sensitivity and adaptive capacity components, Table S5: Index value of different sub-dimensions of exposure, sensitivity and adaptive capacity dimensions, Table S6: Correlation co-efficient; Figure S1: LULC dynamics of the coastal region of Bangladesh during 1998 to 2018, Figure S2: Mapping of susceptibility of agricultural practices to climate change across the coastal districts, Figure S3: Visualization of drivers and buffers of Sensitivity dimension for specific districts on the circumplex charts for planning intervention decision, Figure S4: Visualization of drivers and buffers of adaptive capacity dimension for specific districts on the circumplex charts for planning intervention decision.
