# Supplementary material for: Assessing Agricultural Livelihood Vulnerability to Climate Change in Coastal Bangladesh
Source: Int J Environ Res Public Health. 2019 Nov 18;16(22):4552. doi: 10.3390/ijerph16224552 (PMC6888219; doi:10.3390/ijerph16224552)
Supplement: Supplementary file 1 [file ijerph-16-04552-s001.pdf]

Supplementary

1. Supplementary Concepts, Definition and Methods

1.1. CDI (Crop Diversity Index)

The index is calculated using Shannon's diversity index (Aleksandrova et al. 2016):

$$CDI\ (Shannon\ diversity\ index)_i = -\sum_{j=1}^{S} p_j * ln\, p_j \tag{1}$$

where, i – district in the coastal region, p_j – the proportion of land (ha) used for j specialization, s – total land. The main specialization categories included: cereal crops, pulse crops, oilseed crops, spices and condiments, vegetables, fruits, and industrial crops. Higher ADI suggests more diversity, thus less vulnerability to climate disaster.

1.2. GAP (Gross Agricultural Production)

This indicator shows the per capita gross agricultural production of each district. The source is Bangladesh Bureau of Statistics report 2011 (Jila Batayon). We have considered the production of all 44 different crops (cereal crops, pulse crops, oilseed crops, spices and condiments, vegetables, fruits, and industrial crops), fisheries, livestock, and poultry birds to estimate the GAP.

1.3. AHP (Analytic Hierarchy Process)

The AHP method (Saaty 1980) is one of the most widely used approaches for decision making through alternatives, was applied to weight the indicators. We used an excel based free AHP model of Goepel (2013) that provided with eigenvalues representing the local weight of an indicator (relative importance of an indicator of one level in respect to level immediately above; in our case, sub-components) in a ratio-scale numbers which was then converted as global weight (relative importance of an indicator to the overall goal; in our case, exposure, sensitivity and adaptive capacity). A maximum consistency ratio (CR) of 10% was allowed (Saaty 2012) (Table S2) to ensure the consistency of the measurement in AHP.

Consistency ratio (CR) in AHP was measured as follows:

$$\text{CR}= \frac{CI}{RI} \tag{2}$$

Where CI indicates consistency index and RI represents Random Consistency Index. CI was calculated as follows.

$$\text{CI}= \frac{\delta_{max}-n}{n-1} \tag{3}$$

RI value was collected from Saaty (1980).

2. Supplementary Tables and Figures

Table S1. Random index (RI) values.

n	1	2	3	4	5	6	7	8	9	10
RI	0	0	0.58	0.90	1.12	1.24	1.32	1.41	1.45	1.49

Table S2. Results of AHP analysis.

Determinants	Weight	Primary component	Weight	CR	Indicator	Initial Weight	CR	Final weight
Exposure	1	Climatic change	0.288	0.001	Extreme temperature	0.231	0.040	0.066
					Changes in temperature	0.208		0.060

					Precipitation variability	0.561		0.161
		Disaster events	0.712	0.07	Flood hazard	0.204	0.070	0.145
					Riverbank erosion	0.157		0.112
					Cyclone hazard	0.220		0.156
					Salinity intrusion	0.259		0.185
					Drought intensity	0.161		0.114
Sensitivity	1	Population	0.111	0.049	Population below poverty level	0.227	0.087	0.025
					Dependency ratio	0.247		0.027
					In migration	0.130		0.014
					Rural population	0.132		0.015
					Ethnic population	0.123		0.014
					Female population	0.090		0.010
					Population growth	0.051		0.006
		Health	0.121		Disabled population	0.097	0.027	0.012
					Infant mortality rate	0.108		0.013
					Underweight children	0.083		0.010
					Severely stunted growth	0.087		0.010
					Arsenic problem	0.130		0.016
					Distance from a water source	0.146		0.018
					Unsafe drinking water	0.185		0.022
					Un-hygienic sanitation conditions	0.165		0.020
		Land resources	0.329		Land use intensity	0.353	0.049	0.116
					Land degradation	0.306		0.101
					Soil organic matter	0.136		0.045
					Soil phosphorus	0.205		0.067
		Agricultural practices	0.439		Marginalized farm holdings	0.105	0.087	0.046
					Arable land	0.091		0.040
					Fish-culture area	0.090		0.040
					Rain-fed crop area	0.150		0.066
					Livestock potential	0.113		0.050
					Crop diversity index	0.178		0.078
					Gross agri. production	0.159		0.070
					Productivity of rice	0.114		0.050
Adaptive capacity	1	Human capital	0.159	0.049	Literacy rate	0.243	0.074	0.039
					Youth education	0.263		0.042
					Economically active population	0.309		0.049
					Female work participation	0.186		0.030
		Financial capital	0.182		Income diversification index	0.289	0.029	0.053
					Foreign remitter	0.165		0.030
					Access to farm credit	0.246		0.045
					Share of agricultural GDP	0.215		0.039

		Dependence on agriculture	0.084		0.015
Social and institutional capital	0.165	Farmers associations	0.181	0.091	0.030
		Agricultural markets	0.143		0.024
		Density of schools	0.239		0.039
		Density of healthcare facilities	0.322		0.053
		Rehabilitation support	0.115		0.019
Physical capital	0.244	Structurally sound houses	0.149	0.073	0.036
		Emergency shelters	0.121		0.030
		Road network	0.244		0.059
		Share of embankments/dams	0.194		0.047
		Rural electrification	0.217		0.053
		Use of mobile phones	0.075		0.018
Natural capital	0.072	Open water bodies	0.270	0.072	0.020
		Natural forests	0.300		0.022
		Land potential	0.430		0.031
Agro-technology use	0.175	Adoption of improved crop variety	0.223	0.089	0.039
		Use of fertilizer	0.163		0.029
		Use of pesticide	0.170		0.030
		Irrigation pump	0.180		0.032
		Crop harvester/thresher	0.152		0.027
		Use of bio-gas	0.111		0.020

Table S3. Land cover classification scheme used in this study.

Type	Description
Agricultural land	Cultivated land, crop fields, fallow lands, and vegetable fields
Mixed forest vegetation	Land covered with woodland, trees in the terrestrial forests, around homesteads, along the roadside and crop fields
Mangrove forest	Sundarban and other wetland covered with dense forests both natural and planted
Bare land	Exposed soils, construction sites, mudflat
Built-up land	Residential, commercial and services, industrial, transportation, roads, mixed urban, and other urban
Waterbody	River networks, canals, and active hydrological features
Aquaculture/Salt	Includes water body with saline water excluding major rivers used for shrimp culture or salt production

Table S4. Ranking of coastal districts based on the overall vulnerability index score along with exposure, sensitivity and adaptive capacity components.

District	Exposure index		Sensitivity index		Adaptive capacity index		ALVI	
	Score	Rank	Score	Rank	Score	Rank	Score	Rank
Bhola	0.651	1	0.573	6	0.342	18	0.882	1
Patuakhali	0.549	6	0.655	1	0.389	14	0.816	2
Noakhali	0.524	8	0.542	8	0.306	19	0.759	3

Barisal	0.560	5	0.604	2	0.448	7	0.716	4
Barguna	0.590	3	0.502	13	0.406	13	0.687	5
Lakshmipur	0.413	12	0.604	3	0.349	17	0.668	6
Bagerhat	0.534	7	0.521	9	0.407	12	0.647	7
Cox'sbazar	0.486	9	0.515	10	0.383	15	0.618	8
Chittagong	0.588	4	0.504	12	0.487	3	0.605	9
Chadpur	0.395	13	0.600	4	0.451	6	0.543	10
Pirojpur	0.357	15	0.596	5	0.421	11	0.531	11
Khulna	0.625	2	0.458	14	0.562	1	0.521	12
Feni	0.418	11	0.511	11	0.453	5	0.476	13
Jhalokati	0.325	18	0.562	7	0.426	10	0.460	14
Satkhira	0.470	10	0.453	16	0.473	4	0.450	15
Shariatpur	0.359	14	0.435	17	0.373	16	0.421	16
Gopalganj	0.334	16	0.454	15	0.430	9	0.358	17
Narail	0.328	17	0.428	18	0.432	8	0.324	18
Jessore	0.230	19	0.419	19	0.508	2	0.141	19

Table S5. Index value of different sub-dimensions of exposure, sensitivity and adaptive capacity dimensions.

District	CC	DE	Population	Health	Land	Agriculture	HC	FC	SIC	PC	NC	UAT
Bagerhat	0.202	0.332	0.050	0.062	0.157	0.252	0.082	0.086	0.081	0.055	0.057	0.047
Barguna	0.238	0.352	0.041	0.057	0.205	0.200	0.082	0.091	0.099	0.084	0.032	0.018
Barisal	0.132	0.429	0.059	0.034	0.223	0.288	0.083	0.094	0.081	0.119	0.018	0.052
Bhola	0.147	0.504	0.058	0.052	0.177	0.287	0.038	0.098	0.068	0.042	0.041	0.054
Chadpur	0.152	0.243	0.061	0.059	0.217	0.263	0.062	0.099	0.069	0.116	0.007	0.100
Chittagong	0.188	0.400	0.021	0.021	0.261	0.201	0.121	0.091	0.047	0.120	0.014	0.095
Cox'sbazar	0.109	0.377	0.082	0.040	0.215	0.179	0.044	0.096	0.023	0.068	0.021	0.130
Feni	0.165	0.254	0.043	0.025	0.220	0.223	0.057	0.097	0.043	0.148	0.001	0.106
Gopalganj	0.146	0.188	0.058	0.032	0.185	0.178	0.066	0.098	0.113	0.038	0.010	0.105
Jessore	0.217	0.013	0.041	0.030	0.159	0.189	0.096	0.106	0.054	0.102	0.004	0.147
Jhalokati	0.208	0.117	0.052	0.025	0.208	0.277	0.083	0.102	0.095	0.097	0.008	0.040
Khulna	0.218	0.407	0.020	0.038	0.169	0.231	0.115	0.098	0.105	0.128	0.051	0.065
Lakshmipur	0.037	0.376	0.060	0.050	0.219	0.274	0.028	0.082	0.046	0.109	0.014	0.069
Narail	0.217	0.111	0.038	0.033	0.166	0.191	0.075	0.104	0.059	0.064	0.012	0.118
Noakhali	0.104	0.419	0.052	0.052	0.212	0.225	0.028	0.081	0.024	0.084	0.021	0.069
Patuakhali	0.132	0.416	0.051	0.052	0.237	0.315	0.076	0.092	0.086	0.064	0.035	0.036
Pirojpur	0.187	0.169	0.051	0.060	0.200	0.285	0.083	0.106	0.097	0.084	0.012	0.039
Satkhira	0.145	0.325	0.044	0.060	0.146	0.203	0.086	0.092	0.065	0.093	0.047	0.089

Shariatpur	0.146	0.213	0.069	0.035	0.189	0.143	0.053	0.106	0.052	0.074	0.012	0.076

CC = Climatic change; DE= Disaster events; HC= Human capital; FC= Financial capital; SIC= Social and Institutional capital; PC= Physical capital; NC= Natural capital; UAT =Use of agro technology.

Table S6. Correlation co-efficient.

Variables	Measure	ALVI	EI	SI	ACI
ALVI	Pearson Correlation	1	0.833 **	0.620 **	–0.524 *
	Sig. (2-tailed)		0.000	0.005	0.021
	N	19	19	19	19
Exposure Index (EI)	Pearson Correlation	0.833 **	1	0.240	−0.093
	Sig. (2-tailed)	0.000		0.322	0.706
	N	19	19	19	19
Sensitivity Index (SI)	Pearson Correlation	0.620 **	0.240	1	−0.284
	Sig. (2-tailed)	0.005	0.322		0.239
	N	19	19	19	19
Adaptive Capacity Index (ACI)	Pearson Correlation	−0.524 *	−0.093	−0.284	1
	Sig. (2-tailed)	0.021	0.706	0.239	
	N	19	19	19	19

** Correlation is significant at the 0.01 level (2-tailed); * Correlation is significant at the 0.05 level (2-tailed).

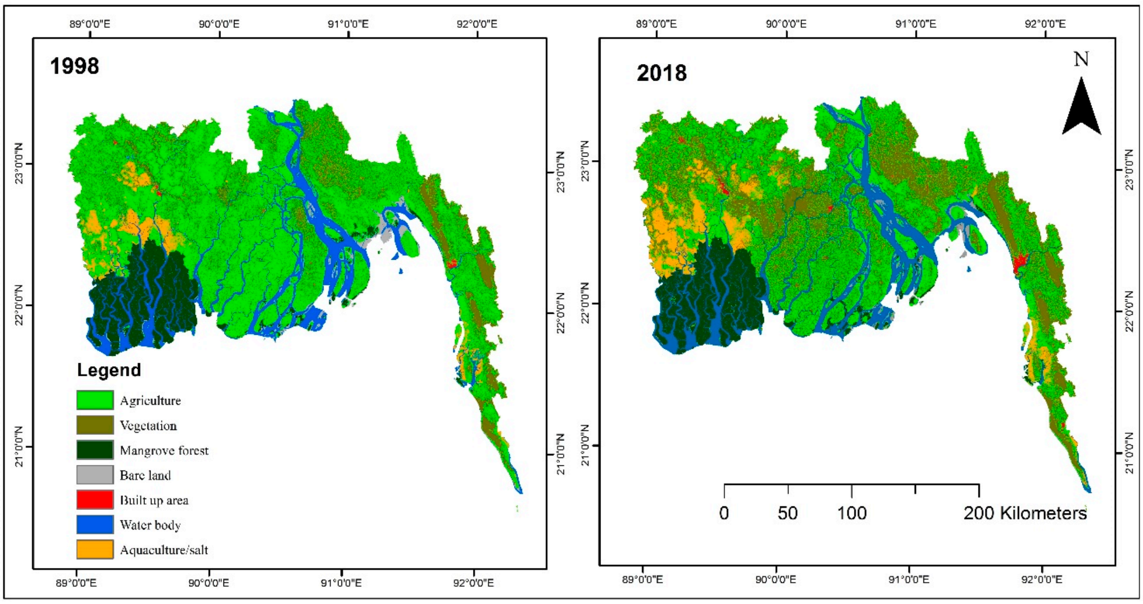

Figure S1. LULC dynamics of the coastal region of Bangladesh during 1998 to 2018.

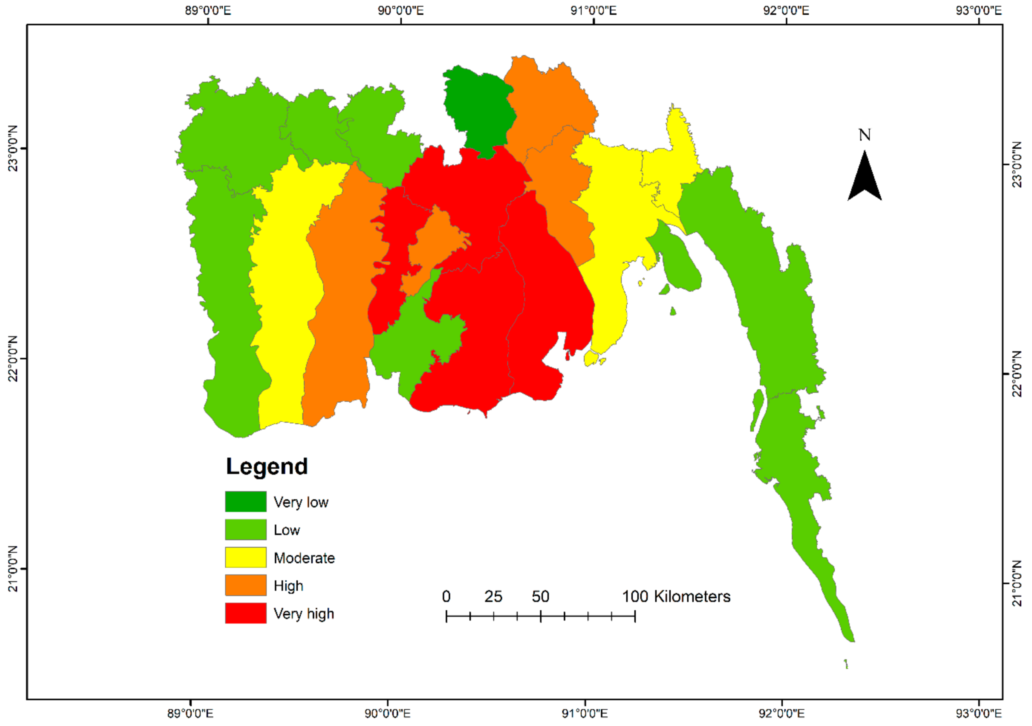

Figure S2. Mapping of susceptibility of agricultural practices to climate change across the coastal districts.

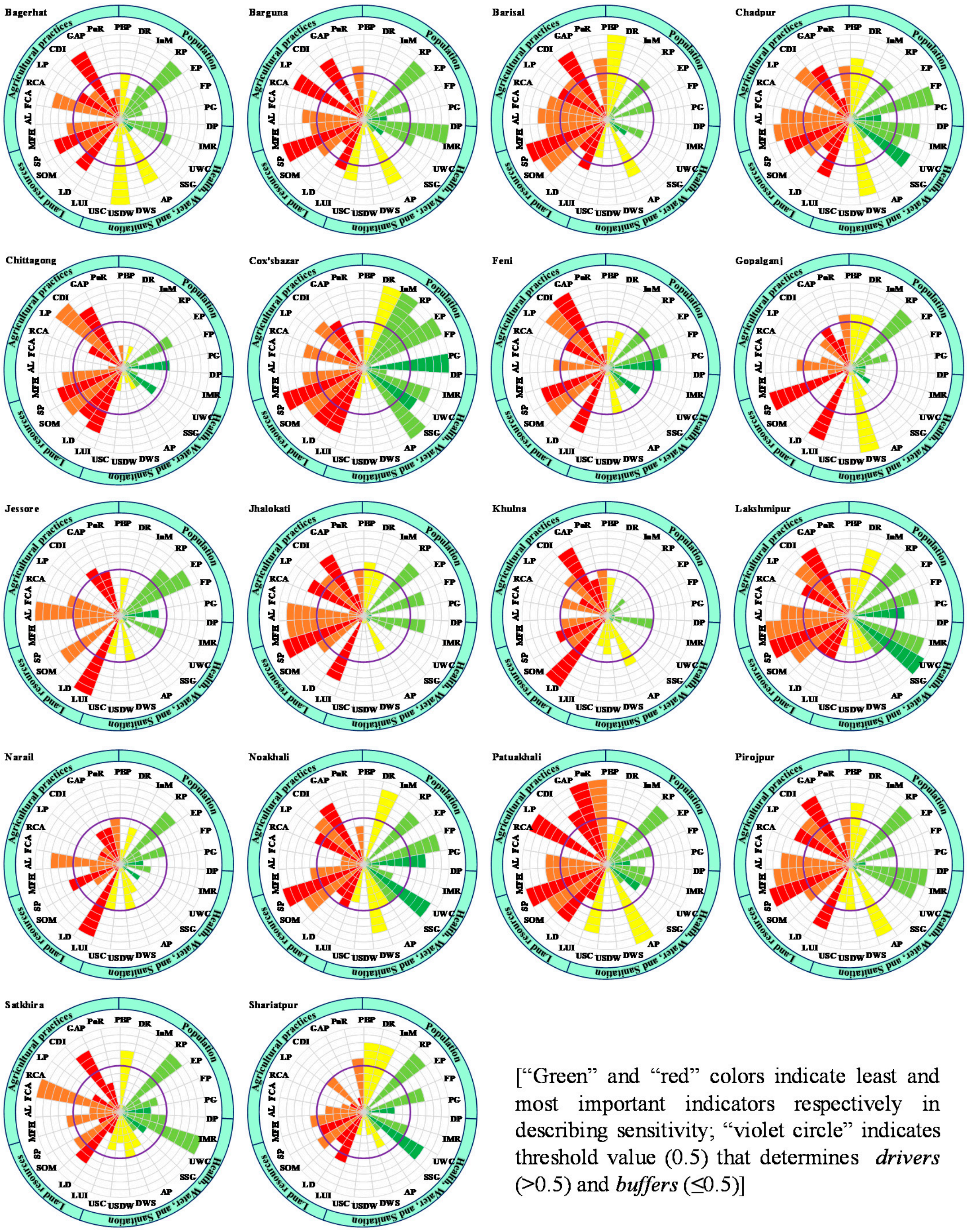

[“Green” and “red” colors indicate least and most important indicators respectively in describing sensitivity; “violet circle” indicates threshold value (0.5) that determines *drivers* (>0.5) and *buffers* (≤0.5)]

Figure S3. Visualization of *drivers* and *buffers* of Sensitivity dimension for specific districts on the circumplex charts for planning intervention decision.

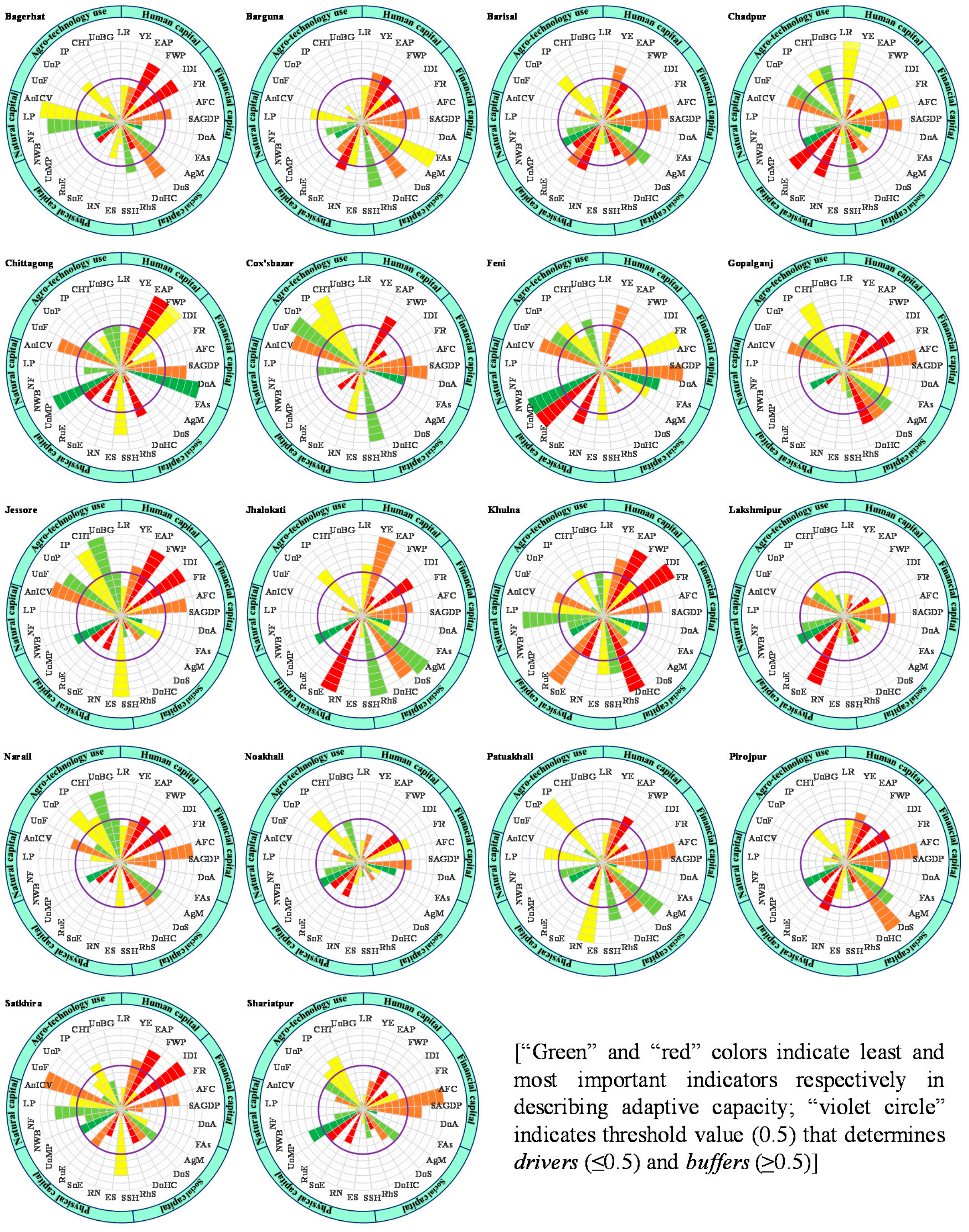

Figure S4. Visualization of *drivers* and *buffers* of adaptive capacity dimension for specific districts on the circumplex charts for planning intervention decision.

References

1. Aleksandrova, M.; Gain, A.K.; Giupponi, C. Assessing agricultural systems vulnerability to climate change to inform adaptation planning: an application in Khorezm, Uzbekistan. *Mitigation and adaptation strategies for global change* **2016**, *21*, 1263–1287.
2. FAO. *Increasing Resilience of Agricultural Livelihoods*; Food and Agricultural Organization of the United Nations: Rome, Italy, 2016.

3. Goepel K.D. Implementing the analytic hierarchy process as a standard method for multi-Criteria decision making in corporate enterprises? A new AHP excel template with multiple inputs. Proceedings of the international symposium on the analytic hierarchy process, Kualalampur. Available online: https://bpmsg.com/new-ahp-excel-template-with-multiple-inputs/ (accessed on 6 December 2017)
4. Saaty, T.L. *The Analytic Hierarchy Process*; McGraw-Hill: New York, NY, USA, 1980.
5. Saaty, T.L. Decision Making for Leaders: The Analytic Hierarchy Process for Decisions in a Complex World, 3rd ed.; RWS Publications: Pittsburgh, PA, USA, 2012.
